# Potential Lack of Association Between Three Vestigial Muscles in Humans: A Willed Body Donor Study

**DOI:** 10.7759/cureus.8098

**Published:** 2020-05-13

**Authors:** Blake H Hodgens, Matthew J McSoley, Jacob E Milner, Kunal P Naik, Kaleb R Howard, Ean Schwartz, David P Matichak, Thomas H Champney

**Affiliations:** 1 Cell Biology, University of Miami Miller School of Medicine, Miami, USA

**Keywords:** vestigial, muscle, human, anatomy, orthopedics

## Abstract

Purpose: Anatomical agenesis within a population is not well understood, with variations including but not limited to complete absence, unilateral presence, or bilateral presence. Agenesis of human vestigial muscles including the palmaris longus (PL), fibularis tertius (FT), and psoas minor (PM) has been studied; however, the relationship between their presence and absence has not been examined. The purpose of this study is to analyze the prevalence of the PL, FT, and PM muscles, investigate any relationship of prevalence based on sex or race, and investigate any correlation between the presence or absence of each muscle within individual donors.

Methods: Twenty-three willed body donors were comprehensively dissected by medical students, and the presence or absence of the PL, FT, and PM muscles was recorded.

Results: The PL was present bilaterally in 87% of donors and absent bilaterally in 13%. The FT was present bilaterally in 96% of donors, and present unilaterally in 4% of donors. There was no evidence of total agenesis of the FT within our sample. The PM was present bilaterally in 39% of donors, and absent bilaterally in 61% of donors. No statistically significant relationship was indicated between muscle presence and the sex or race of the donors. No correlation between the presence or absence of each of the muscles was found.

Conclusion: In this sample of willed body donors, there was no relationship between muscular agenesis of the vestigial muscles. This suggests that muscle agenesis is a local genetic developmental event at each muscle, and that there is not a single developmental event that leads to agenesis of multiple vestigial muscles. Further understanding of the agenesis of vestigial structures within populations and subpopulations can aid in physician diagnosis and understanding of the anatomical makeup of individuals.

## Introduction

Anatomical muscular agenesis within a population is not well understood, with variations including complete absence, unilateral presence, or bilateral presence of the muscles. Agenesis is common in human vestigial structures, which are structures with varying degrees of prevalence and functionality within a population due to the course of evolution [1]. Correlative studies are often performed to assess the relationship between the degree of vestigiality of a particular muscle and demographic parameters. Investigation of the prevalence of vestigial structures within a subpopulation is frequently performed with a clinical, intraoperative, or cadaveric lens.

The palmaris longus (PL) muscle is an anterior forearm muscle that originates with the common flexor tendons at the medial epicondyle of the humerus, coursing inferolateral between the flexor carpi radialis muscle, laterally, and flexor carpi ulnaris muscle, medially, to insert into the flexor retinaculum and palmar aponeurosis on the palmar side of the hand [2,3]. From a gross anatomical perspective, the PL muscle is a long, slender, spindle-shaped fusiform muscle with a small muscular belly and long, thin tendon [3,4]. The PL muscle is innervated by the median nerve as it travels along the anterior side of the forearm, distal to the antecubital fossa. The PL muscle aids in tensing the palmar aponeurosis and therefore functions in corrugating the skin of the palm. The extent of functional vestigiality of the PL muscle is highlighted by (1) a lack of diminished function of the wrist and forearm after intraoperative injury to the PL muscle and (2) congenital absence showing no functional loss of grip or finger flexion strength [2,3]. The tendon of the PL, if present, is commonly used as an autograft in various orthopedic, plastic, and other reconstructive surgeries [3]. Despite common literature stating an absence of 15%, the PL muscle has the greatest variability in prevalence of any muscle in the human body, with absence ranging from 2% to 64%, with a direct correlation with ethnicity [4]. Morphological agenesis of the PL muscle between the sexes has been inconclusive [4]. Through a meta-analysis, there appear to be inconclusive results in regards to unilateral versus bilateral presentation of the PL muscle. Similarly, regarding unilateral agenesis, there is no statistically significant preference for left- or right-sidedness [4].

The fibularis tertius (FT) muscle, also commonly referred to as the peroneus tertius, is located in the anterior compartment of the leg originating from the distal fibula and tendons of the extensor digitorum longus muscle, traveling inferolateral to insert on the base of the fifth metatarsal. The FT muscle is commonly referred to as the extensor digitorum longus “fifth tendon” due to its intimate relationship [5]. The FT muscle receives innervation from the deep fibular nerve, a branch of the common fibular nerve that arises from the sciatic nerve. The FT muscle functions to weakly dorsiflex and evert the foot, sharing a split function with the anterior and lateral leg compartment muscles. Due to its usefulness as an autologous tendon graft, the identification of the FT muscle within a patient can be very important for foot surgeons [6]. The FT muscle gained clinical attention when it was determined arthroscopically to be the landmark reference structure for the anterolateral portal as well as a potential etiology for fractures of the base of the fifth metatarsal bone, including Jones fractures [7]. A large meta-analysis finds the weighted prevalence of the FT muscle in 93% of cadaveric subjects [8].

The psoas minor (PM) muscle is located in the posterior abdominal wall contributing to the iliopsoas musculotendinous unit, along with the psoas major and iliacus muscles [9]. The PM muscle resides anterior to the psoas major muscle, originating from the lateral aspect of the bodies of the 12th thoracic and 1st lumbar vertebrae, coursing inferiorly to insert on the iliopectineal line of the pelvis. The PM muscle receives innervation from the L1 nerve root [10]. Bilateral contraction of the PM muscle results in weak forward flexion of the spine, while unilateral contraction results in ipsilateral bending of the spine [10]. The PM muscle has an intimate relationship with retroperitoneal neurovascular structures, including the genitofemoral nerve, as it pierces through the psoas major muscle and travels inferiorly to innervate the skin on the anterior medial thigh and the cremaster muscle [11,12]. Of the muscles investigated in this study, the PM muscle has the highest incidence of agenesis, present in only 33%-52% of the population [10,11]. The PM muscle has been shown to be absent unilaterally, but the frequency of unilateral agenesis is not well understood [13]. A large disparity of agenesis of the PM muscle exists in regards to race, with agenesis reported as 13% in White men compared to 91% in Black men [14]. Understanding the prevalence of the PM muscle in the population is important for consideration of various clinical syndromes including PM syndrome and psoas compartment syndrome [11-13].

The aims of this study are to (1) determine the presence or absence of the PL, FT, and PM muscles in deceased individuals, (2) determine any relationship between the presence or absence of these muscles and demographic variables including sex and race, and (3) determine any correlation between the presence or absence of these muscles within and between individuals.

## Materials and methods

This study was performed in the anatomical dissection laboratory located at the University of Miami Miller School of Medicine in October 2019, on a total of 23 donors with varying sex, age, and race. Donor characteristics were provided by the Anatomical Board of the State of Florida. No Institutional Review Board (IRB) application was developed, as IRBs in the United States do not review or approve research on deceased individuals. The willed body donors are acknowledged fully in the acknowledgement section and without their generosity and altruism this study could not have been completed. Anatomical dissections were performed by seven medical students, and the presence and/or absence, both unilateral and bilateral, of the PL, FT, and PM muscles was recorded by all seven in an independent fashion. If discrepancies in assessments were found, a group assessment was performed before reporting the final decision. Statistical analysis was performed using the SAS University Edition, SAS Studio 3.8 (SAS Institute Inc., Cary, NC).

## Results

The donor demographics are outlined in Table 1. There were 11 female donors and 12 male donors. With respect to race, there were 17 White, 4 Hispanic/White, and 2 Black donors observed in the study. The mean donor age was 80 years with a range of 45-98 years.

**Table 1:** Demographics of willed body donors

	Total	Male	Female
Donors (n, %)	23 (100%)	12 (52%)	11 (48%)
Age (mean +/- SD)	80 +/- 14	79 +/- 15	86 +/- 12
Age (range)	45-98	45-94	60-98

Race/ethnicity	
White (n, %)	17 (74%)
Hispanic White (n, %)	4 (17%)
Black (n, %)	2 (9%)

Muscle prevalence - total

The PL muscle was present bilaterally in 87% (n=20) of donors and absent in 13% (n=3). It was not present unilaterally in any of the donors. The FT muscle was present bilaterally in 96% (n=22) of donors and unilaterally in 4% (n=1) of donors. No donors were found to have total agenesis of the FT muscle in our sample. The PM muscle was found bilaterally in 39% (n=9) of donors and absent in the remaining 61% (n=14) of donors (Table 2). The FT muscle was the only muscle in our study with unilateral presence.

**Table 2:** Vestigial muscle prevalence in willed body donors Each cell contains the number (and percentage) of willed body donors with or without the indicated muscle.

Muscle		Total (n=23)	Males	Females	White (n=17)	Hispanic/White (n=4)	Black (n=2)
Palmaris longus	Present	20 (87%)	10 (91%)	10 (90%)	16 (94%)	2 (50%)	2 (100%)
	Absent	3 (13%)	1 (9%)	1 (10%)	1 (6%)	2 (50%)	0 (0%)
Fibularis tertius	Present (bilateral)	22 (96%)	11 (92%)	11 (100%)	16 (94%)	4 (100%)	2 (100%)
	Present (unilateral)	1 (4%)	1 (8%)	0 (0%)	1 (6%)	0 (0%)	0 (0%)
	Absent	0 (0%)	0 (0%)	0 (0%)	0 (0%)	0 (0%)	0 (0%)
Psoas minor	Present	9 (39%)	3 (25%)	6 (55%)	7 (41%)	2 (50%)	0 (0%)
	Absent	14 (61%)	9 (75%)	5 (45%)	10 (59%)	2 (50%)	2 (100%)

Muscle prevalence - sex

The PL muscle was present bilaterally in 91% (n=10) of male donors and absent in 9% (n=1) of male donors. The PL muscle was present bilaterally in 83% (n=10) of female donors and absent in 17% (n=2) of female donors. The FT muscle was present bilaterally in 92% (n=11) of male donors and unilaterally in 8% (n=1) of male donors. The FT muscle was present bilaterally in 100% (n=11) of female donors. The PM muscle was present bilaterally in 25% (n=3) of male donors and absent in 75% (n=9) of male donors. The PM muscle was present bilaterally in 55% (n=6) of female donors and absent in 45% (n=5) of female donors (Table 2).

Muscle prevalence - race

The PL muscle was present bilaterally in 94% (n=16) and absent in 6% (n=1) of White donors. The PL muscle was present bilaterally in 50% (n=2) and absent in 50% (n=2) of Hispanic White donors. The PL muscle was present bilaterally in 100% (n=2) of Black donors. The FT muscle was present bilaterally in 94% (n=16) and unilaterally in 6% (n=1) of White donors. The FT muscle was present bilaterally in 100% (n=4) of Hispanic White donors. The FT muscle was present bilaterally in 100% (n=2) of Black donors. No donors had total agenesis of the FT muscle with respect to race in our sample. The PM muscle was present bilaterally in 41% (n=7) and absent in 59% (n=10) of White donors. The PM muscle was present bilaterally in 50% (n=2) and absent in 50% (n=2) of Hispanic White donors. The PM muscle was absent in 100% (n=2) of Black donors (Table 2).

Muscle correlation

The only statistically significant relationship was found between the FT and PL muscles (p<0.05) (Table 3). Statistically significant differences or outcomes simply address whether to accept or reject a null or directional hypothesis, without providing information on the magnitude or direction of the difference or clinical significance. Due to the limited sample size, we recommend caution in drawing any practical clinical conclusions.

**Table 3:** Pearson correlation coefficients and associated p-values between vestigial muscles in willed body donors Each cell contains the Pearson correlation coefficient (and p-value) between the column subject and the row subject in 23 willed body donors.

	Palmaris longus	Psoas minor	Fibularis tertius	Race	Sex
Palmaris longus	1.00	0.05 (.8349)	-0.55 (.0065)	-0.19 (0.3724)	-0.095 (0.6594)
Fibularis tertius	-0.55 (0.0065)	-0.17 (0.4355)	1.00	-0.12 (0.5945)	0.20 (0.3502)
Psoas minor	0.046 (0.8349)	1.00	-0.17 (0.4355)	-0.16 (0.4884)	-0.30 (0.1608)
Race	-0.20 (0.3724)	-0.16 (0.4884)	-0.12 (0.5945)	1.00	0.11 (0.6059)
Sex	-0.09 (0.6594)	-0.30 (0.1608)	0.20 (0.3502)	0.11 (0.6059)	1.00

## Discussion

The prevalence of vestigial structures, including the PL, FT, and PM muscles, is widely variable across individuals. It is important, however, to establish their relative presence, functional importance, and correlative factors to help determine their clinical and surgical application. To gauge and substantiate the data that we have collected from our samples, it is essential to assess how the data of this study compares to that of other studies. It is valuable to gather insight from previously conducted experiments to further hone and expand this area of investigation.

One of the objectives of our study was to analyze the correlative effect that sex has on the presence of the PL, FT, and PM muscles. As indicated in Table 3, sex was found to have no significant effect on the presence of these three vestigial muscles. For the PL muscle, the current literature seems to be inconclusive regarding the role that sex plays in muscle prevalence [4]. In regards to the FT muscle, the literature also seems to have contrasting conclusions with sample size and ethnicity serving as potential confounding variables. Studies with larger sample sizes (n>450) report no significant correlation while studies using smaller sample sizes have reported a higher FT muscle prevalence in males [7]. Considering our results in comparison with the existing literature, additional studies with larger sample sizes would be desired. There is currently no literature regarding the influence of sex on the prevalence of the PM muscle. This study’s findings on this particular topic can serve as preliminary observations for this muscle.

The second objective of this study was to analyze the prevalence of the PL, FT, and PM muscles as they relate to race. Based on the results in Table 3, there was no statistically significant correlation between either of the three racial designations and any of the three vestigial muscles. Contrarily, a comprehensive meta-analysis conducted by Ioannis and colleagues regarding the presence of the PL muscle within a variety of unique racial populations concluded that (1) Black populations display a significantly lower prevalence of aggregate PL muscle agenesis and (2) Caucasian and Hispanic racial populations display much higher percentages of PL muscle agenesis [4]. Similarly, a meta-analysis involving 35 separate studies on 7601 legs supported the high observed prevalence of the FT muscle within our sample and contrastingly indicated that demographic factors like ethnicity and race carry a significant correlation with vestigial muscles [8]. After comparing various ethnic/racial subpopulations, variability in the FT muscle prevalence was observed ranging from 49% within a homogeneous Chilean sample, to 100% within a Bolivian sample [8]. Multiple studies have also highlighted the discrepancy in the prevalence of the FT muscle between Caucasian and Black populations [8]. Despite the limited literature on the presence or absence of the PM muscle in relation to race, one study showed a dramatic difference of agenesis between White males and Black males, at 13% and 91%, respectively [14].

Our final study objective was to analyze whether the presence of an individual muscle correlates with the presence of the other vestigial muscles within an individual donor. Our findings indicate that there is a statistically significant correlation of the PL and FT muscle presence (p=0.0065) within an individual donor. Other within-body correlations were not statistically significant (Table 3). There is limited literature currently available on these within-body relationships and this would be an excellent area to study in the future. The novel correlation between the PL and FT muscles presents the possibility of an interdependence in the development of these two muscles.

This finding, along with the aforementioned findings regarding race and sex, may have implications in surgical and diagnostic settings. The PL muscle has widely been used as a tendon autograft in surgery, as its loss is unremarkable and does not lead to any functional deficits [15]. Similarly, the FT muscle can be an effective autologous tendon graft [16]. Studies have even shown that the median nerve can be mistakenly harvested when grafting the PL tendon; therefore, accurate recognition of the absence or presence of the PL tendon can lead to improved surgical outcomes and a reduction in adverse events [17]. This study, along with other similar studies can help surgeons use patient race, sex, and vestigial relations to accurately identify agenesis and optimize surgical technique and efficacy.

Similarly, both the PL and PM muscles have clinical implications, as they can both present with various clinical syndromes where accurate recognition of their presence or absence may be useful in generating a differential diagnosis. The PL muscle can compress the adjacent median nerve, leading to a neuropathy similar to carpal tunnel syndrome [3]. PM syndrome is characterized by a high tone in the PM tendon that may present with symptoms similar to appendicitis, diverticulitis, or extrauterine pregnancy [11]. The PM muscle may also be involved in psoas compartment syndrome due to its location in relation to neurovascular bundles in the posterior abdominal wall [11]. By using a patient’s sex and demographics, a physician may be able to more accurately predict the presence of the PM muscle to help generate a differential diagnosis when presented with symptoms resembling PM syndrome or psoas compartment syndrome. Although the present study failed to find any significant correlations for PM muscle presence or absence, this study suggests an area of further investigation that may help with clinical and surgical improvements.

It is also possible that embryological or genetic factors play a role in anatomical agenesis. At this time, the embryological origins of the PL, FT, and PM muscles have not been investigated [3,9]. The lack of an interrelationship between the agenesis of the PL, FT, and PM muscles may suggest a unique developmental pattern of the individual muscles as opposed to a single coordinated developmental process. The PL, FT, and PM muscles, as part of the body and limb skeletal musculature, are derived from the dorsal part of the somite, known as the dermomyotome [18]. Primary muscle fibers arise and form the skeletal musculature within the first eight weeks of embryonic development. It is during this period in which many regulatory proteins act on the somite, leading to myogenic differentiation. After eight weeks of development, the majority of muscle fibers are developed in the fetus, and local factors and maternal nutrition predominately play a role in muscle hypertrophy as opposed to hyperplasia [19]. This leads to the hypothesis that the development of skeletal musculature could be dependent on local signaling factors acting on the paraxial mesodermal somites within the embryo. This suggests the possibility that the investigation of local factors may explain agenesis of certain vestigial muscles within individuals despite the presence of other vestigial muscles.

Limitations

This preliminary investigation revealed several key limitations. Our small sample size produced a decreased extrapolatory power. Furthermore, the lack of racial inclusivity/diversity in this study diminished our ability to make conclusions regarding race. There were only three racial designations: White (17), White/Hispanic (4), and Black (2). These obstacles limited our ability to develop further correlative conclusions regarding sex, race, and the inter-relatability of the presence or absence of the PL, FT, and PM muscles.

As a preliminary anatomical study, this investigation serves as a framework for further research. Despite the novel finding of an interrelationship between the agenesis of the PL and FT muscles, more samples must be collected to further substantiate this finding. In an attempt to increase sample size and racial representation, future partnerships with other medical institutions are desirable.

## Conclusions

The present study found no interrelationships between the presence or absence of the PL, FT, or PM muscles in 23 willed body donors. The development of these vestigial muscles appears to occur independently and does not appear to correlate with sex or race. Anatomical agenesis is common among human vestigial muscles. Despite previous research on the topic, relationships between muscle agenesis and individual patient characteristics have not been fully investigated. Furthermore, interrelationships between agenesis of muscles within individuals have not been studied and warrant further investigation. Further understanding of the agenesis of vestigial structures within populations and subpopulations can aid in physician diagnosis and understanding of the anatomical composition of specific patients.
